# Patterns of contemporary hybridization inferred from paternity analysis in a four-oak-species forest

**DOI:** 10.1186/1471-2148-9-284

**Published:** 2009-12-07

**Authors:** Alexandru L Curtu, Oliver Gailing, Reiner Finkeldey

**Affiliations:** 1 Forest Genetics and Forest Tree Breeding, Büsgen-Institute, Georg-August University Göttingen, Büsgenweg 2, 37077, Germany; 2 Department of Forest Sciences, Transilvania University Brasov, Sirul Beethoven 1, 500123 Brasov, Romania; 3 School of Forest Resources and Environmental Science, Michigan Technological University, USA

## Abstract

**Background:**

Few studies address the issue of hybridization in a more than two-species context. The species-rich *Quercus *complex is one of the systems which can offer such an opportunity. To investigate the contemporary pattern of hybridization we sampled and genotyped 320 offspring from a natural mixed forest comprising four species of the European white oak complex: *Quercus robur*, *Q. petraea*, *Q. pubescens*, and *Q. frainetto*.

**Results:**

A total of 165 offspring were assigned unambiguously to one of the pollen donors within the study plot. The minimum amount of effective pollen originating from outside the plot varied markedly among the seed parents, ranging from 0.18 to 0.87. The majority of the successful matings (64.1%) occurred between conspecific individuals indicating the existence of reproductive barriers between oak species. However, the isolation was not complete since we found strong evidence for both first-generation (8.4%) and later-generation hybrids (27.5%). Only two out of eight seed parents, belonging to *Q. petraea *and *Q. robur*, showed a high propensity to hybridize with *Q. pubescens *and *Q. petraea*, respectively. Significant structure of the effective pollen pools (*Φ*_*pt *_= 0.069, P = 0.01) was detected in our sample. However, no support was found for the isolation by distance hypothesis. The proportion of hybrids was much higher (79%) in the seed generation when compared to the adult tree generation.

**Conclusion:**

First-generation hybrids were observed only between three out of six possible species combinations. Hybrids between one pair of species preferred to mate with one of their parental species. The observation of first and later-generation hybrids in higher frequency in acorns than in adults might be explained by selection against hybrid genotypes, the history of this uneven-aged forest or past introgression between species.

## Background

Natural hybridization and subsequent introgression provide opportunities for alleles to travel between species within a genus [1]. The presence of new genetic variants causes an increase of genetic diversity and may also bring novel adaptations in the recipient species [2,3]. Most of the studies on plant natural hybridization focused on one pair of species and only in rare cases the occurrence of this phenomenon was investigated in a three or more species context. The hybridization patterns can be more accurately estimated when another related species occurring in the proximity of a two-species hybrid zone are considered [4,5].

The availability of highly polymorphic genetic markers (e.g. microsatellites, AFLPs) and powerful statistical procedures (e.g. Bayesian analysis) has increased our ability to identify first and later-generation hybrids in a wide range of plant species. Among the long-lived species of the temperate zone, poplars [e.g. [6]] and oaks [7] have received much of the attention. Genetic exchange within the genus *Quercus *was suspected for a long time [8] and confirmed by numerous recent studies which used both chloroplast and nuclear genetic markers [9-12]. Hybridization appears to be influenced by the spatial structure of the population [13] and relative frequency of the parental species [4]. However, a recent study that used a set of microsatellite loci to study pairs of populations distributed across the natural range suggested the existence of reproductive isolation rather than high rates of gene flow between *Q. robur *and *Q. petraea *[14].

Paternity analysis studies have shown that wind-pollinated tree species such as oaks can spread their pollen over long distances [15,16]. Despite the high levels of distant gene flow reported, matings occur commonly among neighboring trees determining significant differences in the genetic composition of the effective pollen pools received by each maternal tree in a single year [15] but also by the same maternal trees among reproductive years [17]. Paternity analyses based on microsatellite markers were used for estimating hybridization rates at a fine scale in mixed oak stands [15,18]. Pollination experiments conducted over several years revealed that hybridization between the most common oak species in Europe, *Q. robur *and *Q. petraea *[19], and between a first-generation hybrid and these two parental species [20] is possible. Pollination of *Q. robur *seed trees by *Q. petraea *pollen is usually more successful than the reciprocal cross [19].

Here, we investigate the patterns of actual hybridization in a mixed natural forest comprising all white oak species which occur in the region. The specific objectives of this study were to: (i) find any evidence of interspecific matings (ii) determine the directionality of gene flow between species (iii) quantify the genetic differences between the effective pollen pools and to correlate these differences with the physical distances between the maternal parents (iv) compare the proportion of hybrids present in acorn collections with the proportion of hybrids among the adult trees of the forest.

## Methods

### Species sampled and study area

Oaks are monoecious species, i.e. they have male and female flowers on the same tree. Their annual regularity of flower production contrasts with their irregular fructification, which may range from abundant crops (mast-years) in some years to poor or no crops in others [21]. The distance between abundant crops may reach 6-10 or even more years for some oak species [22] which makes acorn collection very difficult.

The four closely related species included in this study, *Q. robur*, *Q. petraea*, *Q. pubescens *and *Q. frainetto*, respectively, are deciduous trees and belong to section *Quercus *s.s. [white oaks - sensu stricto [23]]. The study plot is a part of the Bejan Oak Reserve (45°51'N, 22°53'E) which is situated in the Carpathian Basin, west-central Romania. More details about the study area are given elsewhere [24].

### Adult sampling

**Figure 1:**
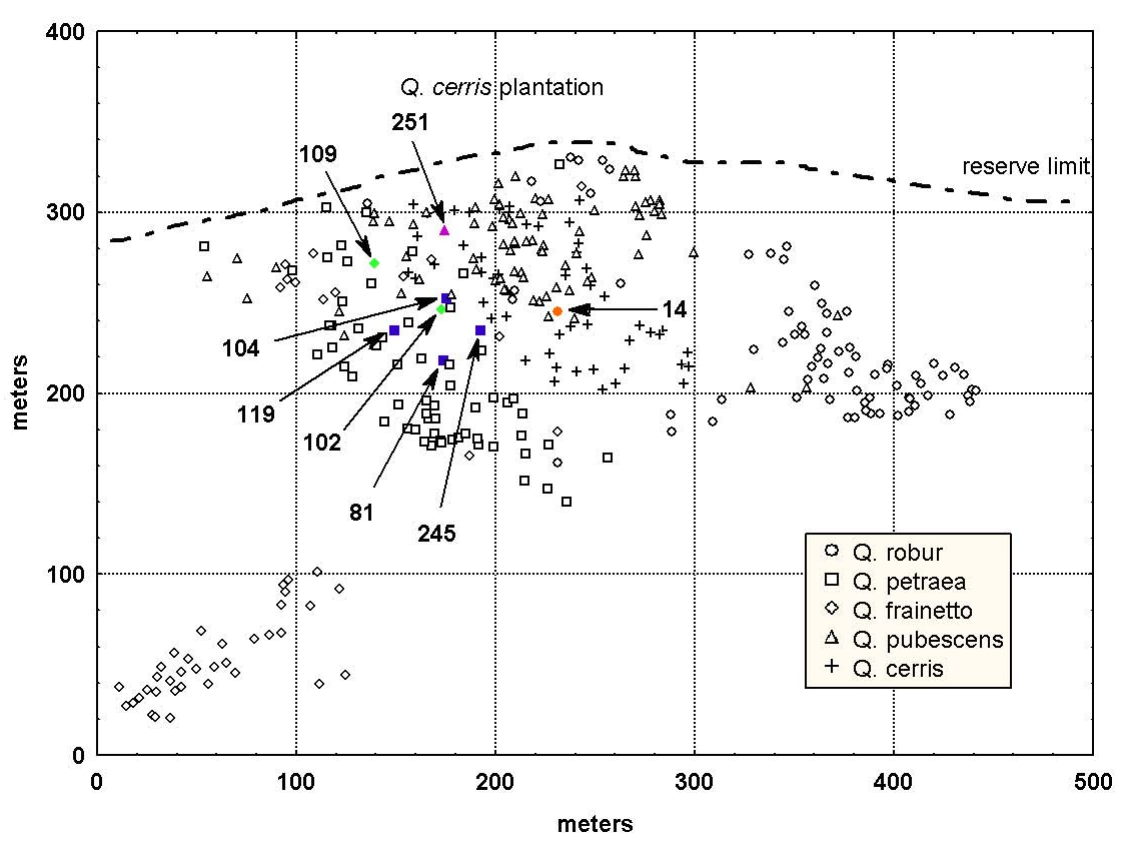
**Location of the adult trees in the study area **[24]. Each species is shown by a different form. The eight maternal trees are shown by filled forms.

A total of 269 adult trees were morphologically and genetically analyzed in a previous study [24]. The trees are about 120-200 yr old and originated from natural regeneration. Most of the individual trees are located within the core plot, an area where the sampling of all white oak trees was exhaustive. As the sample size for *Q. frainetto *within the core plot was very small (15 trees) the sampling for this species was extended to the nearby area (Figure 1). Based on pubescence and leaf descriptors the vast majority of individual trees (~94%) were classified as belonging to a morphological species [24]. Furthermore, based on a set of 6 microsatellite and 7 isozyme markers and using a Bayesian admixture analysis implemented in the program Structure version 2.1 [25] without any *a priori *information on morphology we assigned adult individuals to pure species and hybrids [24]. There was a very good correspondence of each morphological species (e.g. *Q. robur*) with one of the inferred genetic clusters (e.g. '*robur*'). In this study we classified each tree with a high probability to belong to one of the four genetic clusters (admixture coefficient, Q > 0.90) as pure species and Q < 0.90 as hybrid (sensu lato, including introgressed forms). However, as in the recent study of Lepais and his colleagues [4] individuals with Q < 0.90 for one genetic cluster (e.g. '*robur*'), and Q < 0.10 for the remaining three clusters ('*petraea*', '*frainetto*', and '*pubescens*') were considered as pure species.

### Offspring sampling

We genotyped 320 acorns collected in autumn 2005 from eight seed parents: four *Q. petraea*, two *Q. frainetto*, one *Q. robur*, and one *Q. pubescens*, respectively. According to the genetic assignment of the adult tree generation, 7 out of 8 seed trees were classified as pure species [24]. Only one seed parent (Fra-102) was morphologically classified as *Q. frainetto *but genetically was considered as hybrid (sensu lato) between '*frainetto*' and '*pubescens*' clusters. The sampling design was affected by the nearly complete lack of fructification for two species, *Q. robur *and *Q. pubescens*, in 2005 at our study site. All maternal trees are located in the central part of the study plot (Figure 1). The acorns were collected on the ground near the trunk of each seed parent after shooting the crown with a gun.

### DNA isolation and microsatellite typing

Details of the method used for DNA extraction, PCR amplification and microsatellite genotyping of the adult trees are given elsewhere [26]. Using the same procedure the genotypes of the offspring were scored at six microsatellite loci. Loci *ssrQpZAG1*/5, *ssrQpZAG9*, *ssrQpZAG36 *and *ssrQpZAG104 *were originally developed for *Q. petraea *[27], *ssrQrZAG96 *was developed for *Q. robur *[28] and *MSQ13 *for *Q. macrocarpa *[29].

### Statistical data analysis

Paternity analysis (the assignment of a putative father to a genetically known mother-offspring pair) was performed using the likelihood method implemented in the program FAMOZ [30]. A simulation assuming both parents inside and outside the study plot was done using 10, 000 offspring to estimate the threshold for paternity [see details in [31]]. Since mistyping is very likely to occur when scoring microsatellite alleles, the error rate was set to 0.0001 - both in the simulation and in the assignment of the most likely father. The genotype with the highest log (logarithm) of the odds ratio (LOD) score above the threshold value was considered as the most likely pollen parent. The LOD score represents the likelihood of a particular genotype being the father compared to all other genotypes.

The male gametic contribution (pollen haplotype) was inferred by subtracting the female contribution from the offspring genotype. In ambiguous cases, in which the seed parent and its offspring were heterozygotes and shared the same pair of alleles at one locus, the pollen profile was constructed using the maximum likelihood estimator of pollen parent contribution [see details in [32]]. We assumed that an unknown pollen parent from outside the study plot (external pollen) was successful, if none of the investigated trees could produce a pollen with the inferred multilocus paternal haplotype, for example due to the occurrence of an allele in the progeny which was not observed for any adult tree. Successful pollination by external pollen cannot be ruled out even if one or more potential pollen parents within the study plot were identified and the most likely pollen parent was selected based on its LOD score. Thus, the reported proportions of external pollen are minimum estimates. However, strong deviations from these estimates to the 'true' rate of successful external pollen are unlikely due to high exclusion probabilities.

To quantify the genetic differentiation among the effective pollen pools of the seed trees, an analysis of molecular variance (AMOVA) of male haplotypes was performed using the software package GenAlEx version 6.2 [33]. The significance of the *Φ*_*pt*_, an analogous of *F*_*st*_, was tested by 9999 random permutations. This analysis was conducted for the total effective pollen pools and for the separate effective pollen pools originating from inside and outside of the study plot. By using the same software we then tested the isolation by distance hypothesis. The association between the two matrices, pairwise *Φ*_*pt *_between the effective pollen pools of single tree progenies and pairwise physical distances between the maternal parents, was analyzed using the Mantel test statistics [34]. Statistical significance was tested by means of 9999 permutations.

## Results

### Paternity analysis

The six microsatellite markers were highly polymorphic, resulting in high exclusion probabilities for paternity analysis (Table 1). Only a few alleles that were not present in the adult trees were found among the offspring (Table 1). Strong evidence for a null (non-amplified) allele was found at locus *ssrQpZAG1/5 *in *Q. petraea*. Mismatches between one seed parent (Pet-104) and its offspring were observed at this locus whereas the same offspring shared at least one allele with the seed parent at five remaining loci. Moreover, the seed parent as well as all the mismatching offspring appeared to be homozygotes, supporting the hypothesis that both seed parent and mismatching offspring are heterozygous for a null allele. In accordance with the presence of null alleles, the greatest deficit of heterozygotes (*F*_*IS *_= 0.11) amongst the six microsatellite loci in the four oak species was observed at the locus *ssrQpZAG1/5 *in *Q. petraea *adult trees [26].

**Table 1:** Microsatellite data

Locus	N	**n**_**off**_	Size range of alleles (bp)	Exclusion probability
*ssrQpZAG1/5*	32	1	136-192	0.806020
*ssrQpZAG9*	20	1	179-259	0.760460
*ssrQpZAG36*	27	1	198-236	0.789043
*ssrQrZAG96*	24	0	136-182	0.826476
*ssrQpZAG104*	45	1	183-249	0.908133
*MSQ13*	21	1	183-241	0.714140
*Cumulative*	169	5	-	0.999955

Number of alleles in the adult tree generation (n), number of new alleles detected among the offspring (n_off_), size range of alleles and exclusion probability for six microsatellite loci.

**Figure 2:**
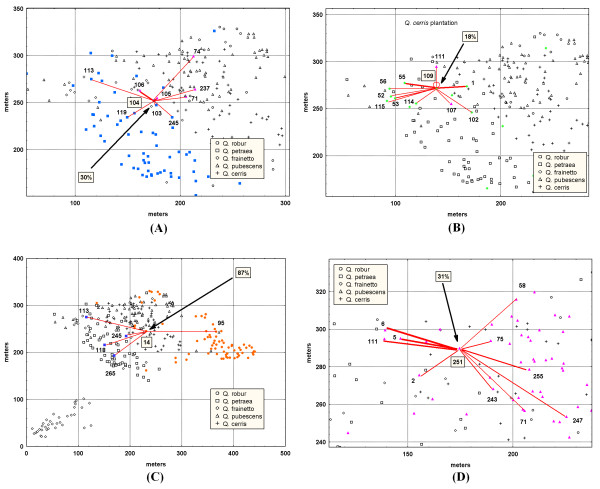
**Spatial distribution of four maternal trees belonging to different oak species (A - *Q. petraea*, B - *Q. frainetto*, C - *Q. robur *and D - *Q. pubescens*, respectively) and their pollen donors within the study plot**. Each pollen donor is linked to the corresponding maternal tree through a line. The thickness of each line is proportional to the number of successful matings. The proportion of pollen coming from outside the study plot is also given. Each species is shown by a different shape and colour. Phenotypically intermediate individuals are indicated by the symbols of the species they mostly resemble.

The pollen donors that were assigned to the offspring are shown exemplarily in Figure 2A-D for four maternal trees, one of each oak species. Among 165 offspring having their pollen donor within the study plot only three appeared to be self-pollinated. Averaged across all offspring and seed parents, selfing rate was very low (about 1%) and due to selfing in one single *Q. frainetto *seed parent (Figure 2B).

Mean pollen dispersal distances are given for each seed parent in Table 2. The observed values differ considerably among seed parents ranging from 20.9 m to 92.2 m. Spatial distribution of each oak species within the plot, e.g. the presence or absence of conspecific trees in the neighborhood of each seed parent seems to affect markedly the dispersal distances (Figure 2). The gene flow from outside the study plot was estimated at 48% when averaged across all seed parents. However, this rate varied markedly among the seed parents although they are located relatively close to each other (Figure 1). For instance, relatively low rates of apparent gene flow from outside were observed for the two *Q. frainetto *(18% to 24%) and one *Q. pubescens *(31%) maternal parents compared to the *Q. robur *maternal parent (87%). The proportion of gene flow originating from outside substantially varied across *Q. petraea *seed parents (with a range of variation from 30% to 79%).

**Table 2:** Mean and standard deviation of pollen dispersal within the study plot

Maternal tree	Probability to belong to species genetic cluster (Q)	Number of sampled offspring	Number of offspring resulting from matings within plot	Mean pollen dispersal (m)	Standard deviation (m)
Pub-251	0.98	51	35	36.6	10.4
Pet-081	0.93	29	8	24.5	16.9
Pet-104	0.95	27	19	20.9	18.0
Pet-119	0.97	38	18	24.7	12.6
Pet-245	0.97	48	10	60.8	35.9
Fra-102	0.18	45	34	52.6	24.6
Fra-109	0.93	44	36	32.6	7.6
Rob-014	0.92	38	5	92.2	37.2
Total		320	165		

Species names were abbreviated as follows: Rob - *Q. robur*; Pet - *Q. petraea*; Pub - *Q. pubescens*; Fra - *Q. frainetto*.

### Hybridization between oak species

The majority of acorns gathered from the seven pure species seed parents were fertilized by conspecific pollen (84 out of 131, 64.1%). Eleven offspring (8.4%) appear to be first-generation hybrids since their parents belong to different pure species (Q > 0.90). Seven were F_1 _hybrids between *Q. petraea *(female) and *Q. pubescens *(male), three between *Q. robur *and *Q. petraea*, and one between *Q. frainetto *and *Q. pubescens*, respectively. Even though offspring from four pure *Q. petraea *mother trees were investigated, all first-generation hybrids between *Q. petraea *and *Q. pubescens *were detected among the acorns collected from one *Q. petraea *tree, Pet-104 (Figure 2).

The presence of putative hybrids (Q < 0.90) among effective pollen donors was interpreted as introgressive hybridization in 36 out of 131 mating events (27.5%). Putative hybrids between *Q. petraea *and *Q. pubescens *(13 matings, with an averaged Q = 0.58 for the '*petraea*' cluster) and one hybrid between *Q. pubescens *and *Q. frainetto *(Q = 0.05 for the '*petraea*' cluster) fertilized *Q. petraea *mother trees. One hybrid between *Q. petraea *and *Q. pubescens *(Q = 0.33 for the '*pubescens*' cluster) fertilized four ovules of the *Q. pubescens *mother tree. Moreover, one hybrid between *Q. robur *and *Q. pubescens *fertilized seven ovules of the same *Q. pubescens *tree. Three different hybrids between *Q. frainetto *and *Q. pubescens *(10 matings, averaged Q = 0.52 for the '*frainetto*' cluster) were observed among the pollen donors of the *Q. frainetto *seed tree, Fra-109. None of the *Q. frainetto *trees situated outside the core plot (35 out of 50) was found among the effective pollen donors of the *Q. frainetto *seed tree (Figure 1 and 2). One putative hybrid between *Q. petraea *and *Q. robur *(Q = 0.36 for the '*robur*' cluster) fertilized one ovule of the *Q. robur *mother tree.

**Figure 3:**
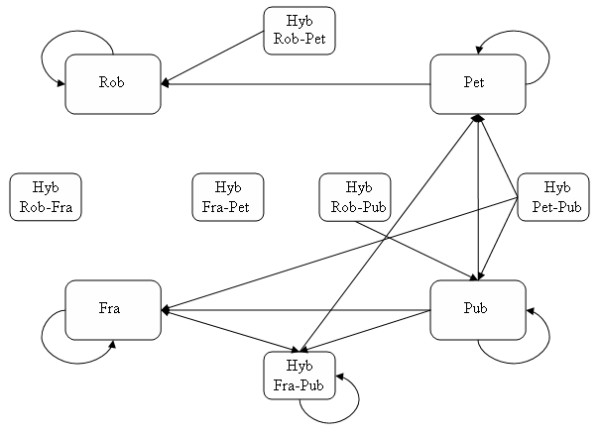
**Pollen flow between four oak species and their hybrids detected in our sample**. The assignment of adult individuals in pure species and hybrid oaks was made by Structure software without any prior information on morphology [24]. Species names were abbreviated as follows: Rob - *Q. robur*; Pet - *Q. petraea*; Pub - *Q. pubescens*; Fra - *Q. frainetto*, Hyb - hybrids between pairs of species.

Among 34 offspring of the single mother tree, Fra-102, which was considered as hybrid between *Q. frainetto *and *Q. pubescens *(Q = 0.18 and Q = 0.76, for '*frainetto*' and '*pubescens*' cluster, respectively) in spite its *Q. frainetto *physical appearance, we detected as pollen donors both pure parental species (65%, 22 *Q. frainetto *and one *Q. pubescens*, respectively) and putative hybrids (11) between this pair of species. The total pollen flow between pure species as well as between pure species and their hybrids, detected in our sample, is illustrated in Figure 3.

### Differentiation among the effective pollen pools

The genetic differentiation (*Φ*_*pt *_= 0.069) among the eight effective pollen pools averaged over all loci was significant (P < 0.05). Pairwise *Φ*_*pt *_values between the effective pollen pools of eight single-tree progenies varied from 0.001 to 0.132 (Table 3). No significant differences were detected among the effective pollen pools of three *Q. petraea *seed parents. Only the effective pollen pool of the highly hybridizing *Q. petraea *tree, Pet-104, differed significantly from the effective pollen pools of other *Q. petraea *and *Q. pubescens *seed trees. Interestingly, there was no significant difference between the effective pollen pools received by the *Q. robur *seed parent, Rob-014, and one *Q. petraea *tree, Pet-081. There was almost no genetic difference between the effective pollen pools of the *Q. frainetto *seed parent and the putative hybrid between *Q. frainetto *and *Q. pubescens *(*Φ*_*pt *_= 0.001) (Table 3).

**Table 3:** Pairwise *Φ*_*pt *_values among the effective pollen pools of eight single-tree progenies

	Rob-014	Pet-081	Fra-102	Pet-104	Fra-109	Pet-119	Pet-245	Pub-251
Rob-014	0							
Pet-081	0.008 ^ns^	0						
Fra-102	0.121***	0.117 ***	0					
Pet-104	0.043***	0.042 ***	0.097 ***	0				
Fra-109	0.120***	0.113***	0.001 ^ns^	0.091 ***	0			
Pet-119	0.011*	0.001 ^ns^	0.112 ***	0.033 ***	0.109 ***	0		
Pet-245	0.012*	0.007 ^ns^	0.131 ***	0.047 ***	0.132 ***	0.007 ^ns^	0	
Pub-251	0.063***	0.046 ***	0.092 ***	0.041 ***	0.078 ***	0.055 ***	0.078 ***	0

Species names were abbreviated as follows: Rob - *Q. robur*; Pet - *Q. petraea*; Pub - *Q. pubescens*; Fra - *Q. frainetto*. ^ns ^P > 0.05; * P < 0.05; ** P < 0.01; *** P < 0.001. P values were estimated after 9999 permutations.

When considering the bulked data over eight seed parents the genetic differences between the effective pollen pools from inside and outside the study plot were significant across all loci (*Φ*_*pt *_= 0.025, P = 0.01). However, at two loci, *ssrQpZAG1/5 *and *ssrQpZAG9*, the differences were not significant (P = 0.16 and P = 0.46, respectively). Our data provided no support for a strong relationship between the pairwise *Φ*_*pt *_values and spatial distances (Mantel test, *R*_*xy *_= 0.041, P = 0.38). Even with regard to the limited sample size this result suggests that spatial distance between the seed parents had no significant impact on the differentiation of the effective pollen pools received by each seed parent.

## Discussion

The present study provides new insights into pollen mediated-gene flow and hybridization within the European white oak species complex. Mismatches between putative parents and offspring due to the presence of a suspected null allele were reported in other studies [e.g. [16]] and seem to be relatively common when employing microsatellite markers in parentage analyses [see [35] for a review]. Selfing was low, as expected in monoecious wind-pollinated tree species of the temperate zone such as oaks [e.g. [15]]. The selfed offspring originate from one *Q. frainetto *seed parent, the species with the lowest density in the area [36]. According to Streiff and her colleagues [15] the effective pollen dispersal in oaks involves two components: local dispersal and long-distance transport. In our study, the portion of local pollen dispersal was quite variable among the seed parents. For instance, the local component of dispersal seems to be prevalent in *Q. frainetto *and *Q. pubescens *seed parents, the species with the lowest relative abundance within the whole forest reserve [36]. In contrast, the amount of pollen originating from outside the study plot was much higher for the three *Q. petraea *seed parents (except for the highly hybridizing seed parent, Pet-104) which is consistent with other results obtained via paternity analyses in different oak species [15,16,37]. According to the forest records, *Q. petraea *is the predominant species in the reserve and in the nearby forest which may explain the larger amount of pollen coming from outside the study plot for this species.

By using paternity analyses we studied pollination patterns in a very complex situation involving all species which occur not only in the mixed stand but also in the nearby area on tens of kilometers. The importance of considering all species when studying hybridization was very well highlighted in France where hybrids were detected in considerable proportions in a mixed stand in the absence of parental species [4]. Our findings suggest the existence of reproductive barriers between oak species since the majority of the effective pollen originates from the same species. However, the hybridization rate was estimated at 35.9% in the seed generation. We found strong evidence for the production of first generation hybrids (8.4%) because both parents were phenotypically and genetically pure species. We observed F_1 _hybrids between three out of twelve possible species combinations, including reciprocal crossings, which suggest that hybridization at this site is not restricted to one species pair (see Figure 3). This result is strongly influenced by our unbalanced sample of mother trees of different species. Previously, in a two-oak-species stand Streiff and her colleagues [15] reported hybridization in 23 out of 310 offspring (7.4%). However, this rate of interspecific gene flow was estimated considering only the physical appearance of oak trees. As in our study the hybridization rate varied markedly among maternal parents. Those oaks showing high levels of hybridization were mostly surrounded by individuals belonging to other species. In such contact zones between species, hybridization may occur due to physical proximity [11]. Hybridization was not restricted to the formation of first-generation hybrids since 27.5% of the pollen donors were putative introgressed forms between the oak species. Nearly 3/4 of the total number of hybrids were later-generation hybrids. The predominance of backcrossed individuals in the detriment of first generation hybrids was reported for the same species complex in western Europe [4,18].

By comparing the overall proportion of hybrids in the adult generation (20.1%, recalculated sensu [4]) with the proportion of hybrids in the offspring (35.9%, without considering the acorns gathered from the single hybrid mother tree) we found nearly 80% more hybrids in the seed generation. The decrease in the number of hybrids from offspring to adults as well as the maintenance of species identity may be the result of the selection against hybrids [38] which acts in the ecological context of this forest. Environmental selection against hybrids was invoked to explain the overestimation of hybridization rates when analyzing seeds [14]. Another explanation for this situation would be the stand history. The uneven-aged structure of the adult population might indicate that some of the trees were established when less than four species were found at this site. The higher proportion of hybrid acorns compared to hybrids adult trees may also be caused by past introgression events which have reduced the strength of the reproductive barriers over time [39].

Our results indicate that hybrids participate at reproduction both as pollen donor and receptor (the case of seed tree Fra-102). Similar situations were reported in a mixed stand with *Q. petraea*, *Q. pubescens *and intermediate individuals between both species in Italy [18]. Moreover, hybrids between one pair of species prefer to mate with one of their parental species. Only in two instances (~3%, 2 out 59 matings between pure species and putative hybrids) we found hybrids which mate with other species than the parental ones. These preferential matings indicate weaker reproductive barriers between the parental species and their hybrids as compared to other related species and hybrid combinations.

The asymmetric gene flow from *Q. petraea *to *Q. robur *revealed by paternity analysis in our sample is consistent with the outcome of the Bayesian analysis in the adult generation [24]. A possible explanation is the contrasting relative abundance of the two species at Bejan: *Q. petraea *is very common in the study area while *Q. robur *is frequent only in a small marginal part of the reserve. Higher success rates of the combination *Q. robur *(female) × *Q. petraea *(male) were also observed in artificial crosses [19,40]. Our finding supports the model of asymmetrical hybridization between *Q. petraea *and *Q. robur *[7]. Moreover, the gene exchange between *Q. pubescens *and *Q. petraea *was mainly from *Q. pubescens *towards *Q. petraea *(20/4 matings) in our sample. The opposite direction was observed between the two species in a hybrid zone in Italy [18]. The ecological conditions and forest composition which differ between the two geographical areas may have influenced the direction of introgression [41]. These discrepancies between hybridization patterns described throughout oaks' distribution range stress the considerable role played by ecological factors and the necessity for comparative studies.

The level of hybridization may be higher for one individual or another because of an overlap in flowering time with most of the individuals of other species. Almost no differences in flowering time between oak species have been reported in different mixed forests [42,43]. For instance, large differences in flowering time between the single *Q. robur *seed parent and the majority of *Q. robur *adult trees and an overlap of the flowering time with most of the *Q. petraea *individuals can be advocated as one of the causes to explain the high propensity for hybridization of this seed tree. Differences in flowering time may lead to a preponderance of pollen coming from *Q. petraea *relative to the pollen coming from its conspecific individuals. Phenological observations at this site are needed to validate this hypothesis. Furthermore, the present results are based on only one flowering season and the patterns of pollen movement may certainly vary from year to year [17]. Hence, the physical barrier represented by *Q. cerris *trees might have been also hindered the pollen coming from the eastern part of the study plot, where *Q. robur *is predominant (Figure 1).

The low amount of genetic differentiation among the effective pollen pools for seed parents which belong to the same species may be due to the limited number of sampled maternal parents. Genetic heterogeneity of effective pollen pools was reported in other studies based on larger sample sizes [15,37]. Genetic heterogeneity of effective pollen pools may be caused by uneven reproductive contribution of local pollen donors [44]. Differences in flowering phenology have also been mentioned as a possible cause [15]. However, the differentiation of effective pollen pools for seed parents which belong to different species was highly significant, except for the effective pollen pool received by the single *Q. robur *seed parent which was very similar to the effective pollen pool of *Q. petraea *trees. The low amount of genetic differentiation between the effective pollen pool received by the *Q. robur *seed parent and the three other *Q. petraea *neighboring trees is consistent with the result of the paternity analysis that indicated a high rate of hybridization with *Q. petraea *trees. In contrast to the study of Streiff and her colleagues [15] a significant differentiation between the effective pollen pools from outside and inside the study plot was detected. This genetic heterogeneity may be caused by the small number of pollen donors identified within the study plot that were not representative for the genetic composition of the whole oak forest. Our data did not follow an isolation by distance pattern like in other tree species [37,45]. The high number of oak species which are present among the sampled maternal trees correlated with their preference for conspecific pollen rather than heterospecific pollen may explain this pattern.

## Conclusion

By using paternity analysis and a set of six highly polymorphic microsatellite loci we have traced patterns of contemporary gene flow within and between oak species in a natural reserve. Our results confirm that introgressive hybridization between different pairs of species is quite common and play a significant evolutionary role within the white oak species complex. Selection against hybrids over years might contribute to the maintenance of species identity despite considerable amounts of interspecific gene flow. However, the differences between the proportion of hybrid acorns and the proportion of hybrid adults may also be explained by the stand history or past introgression. Further phenological investigations and a larger sample size are required for a better understanding of the hybridization events taking place in this four-oak-species stand.

## Authors' contributions

ALC conceived the idea, collected the seed samples, did the laboratory work, performed the paternity analysis, and wrote the majority of the text. OG helped to analyze the data and to draft the manuscript. RF participated in the design and coordination of the study, data interpretation and made substantial contributions to writing this paper.
